# Introducing a change in hospital policy using FMEA methodology as a tool to reduce patient hazards

**DOI:** 10.1186/s13584-016-0090-7

**Published:** 2016-11-01

**Authors:** Fanny Ofek, Racheli Magnezi, Yaffa Kurzweil, Inbal Gazit, Sofia Berkovitch, Orna Tal

**Affiliations:** 1 Medical Center Pharmaceutical Services, Assaf Harofeh Medical Center, Zerifin 70300, Israel, affiliated to the Sackler Faculty of Medicine, Tel Aviv University, Tel Aviv, Israel; 2 Public Health and Health Systems Management Program, Bar Ilan University, Ramat Gan, Israel; 3 Quality Array, Assaf Harofeh Medical Center, Zerifin 70300, Israel, affiliated to the Sackler Faculty of Medicine, Tel Aviv University, Tel Aviv, Israel; 4 Medical Center Pharmaceutical Services Management, Assaf Harofeh Medical Center, Zerifin 70300, Israel, affiliated to the Sackler Faculty of Medicine, Tel Aviv University, Tel Aviv, Israel; 5 Management, Assaf Harofeh Medical Center, Zerifin 70300, Israel, affiliated to the Sackler Faculty of Medicine, Tel Aviv University, Tel Aviv, Israel

**Keywords:** FMEA, Failure mode and effect analysis, Potassium chloride, Quality management, General methodology, Hospital care, Qualitative methods

## Abstract

**Background:**

Intravenous potassium chloride (IV KCl) solutions are widely used in hospitals for treatment of hypokalemia. As ampoules of concentrated KCL must be diluted before use, critical incidents have been associated with its preparation and administration. Currently, we have introduced ready-to-use diluted KCl infusion solutions to minimize the use of high-alert concentrated KCl. Since this process may be associated with considerable risks, we embraced a proactive hazard analysis as a tool to implement a change in high-alert drug usage in a hospital setting.

**Methods:**

Failure mode and effect analysis (FMEA) is a systematic tool to analyze and identify risks in system operations. We used FMEA to examine the hazards associated with the implementation of the ready-to-use solutions. A multidisciplinary team analyzed the risks by identifying failure modes, conducting a hazard analysis and calculating the criticality index (CI) for each failure mode. A 1-day survey was performed as an evaluation step after a trial run period of approximately 4 months.

**Results:**

Six major possible risks were identified. The most severe risks were prioritized and specific recommendations were formulated. Out of 28 patients receiving IV KCl on the day of the survey, 22 received the ready-to-use solutions and 6 received the concentrated solutions as instructed. Only 1 patient received inappropriate ready-to-use KCl.

**Conclusions:**

Using the FMEA tool in our study has proven once again that by creating a gradient of severity of potential vulnerable elements, we are able to proactively promote safer and more efficient processes in health care systems. This article presents a utilization of this method for implementing a change in hospital policy regarding the routine use of IV KCl.

## Background

During the last decade, many Israeli hospitals have joined an accreditation process in order to receive the prestigious quality approval certificate from the Joint Commission International (JCI). This approval confirms that all the medical activities in the hospital are carried out in a safe and coordinated manner and on the basis of the most up-to-date medical guidelines and knowledge available. The accreditation process thoroughly examines different aspects of the hospital’s activities focusing on International Patient Safety Goals (IPSG). IPSG address areas of concern related to patient safety. High-alert drugs constitute one these major areas.

Assaf Harofeh Medical Center is one of Israel’s largest tertiary medical centers, with over 800 acute care beds. It serves a population of over 500,000 in central Israel. The hospital provides all the major services including: emergency, intensive care, general medical, surgical, cardiac, pediatric, neonatal, gynecological and obstetrical medical services. Up to recently, concentrated KCl solutions (14.9 %) were supplied to all hospital wards as part of the routine weekly order. These concentrated solutions were stored in separate cupboards away from other solutions and medications and marked with a special sticker indicating that they are high-alert drugs. The nurses prepared the solution for infusion according to doctors’ orders by inserting the required dose of KCl into the appropriate infusion solution bag (saline, glucose 5 % etc.).

Potassium chloride (KCl) is an electrolyte most commonly used for potassium replacement in various clinical conditions related to hypokalemia (low potassium levels). KCl administration via the intravenous (IV) route should only be used when the oral or enteral route is not possible or will not achieve the required increase in serum potassium within a clinically acceptable time [1–3]. Since severe hypokalemia (<2.5 mEq/L) may result in muscle necrosis and cardiac arrhythmias, concentrated KCl IV solutions are widely used and administered as diluted solutions to treat this condition. These patients may require rapid infusion of IV KCl. A delay in administering this therapy could compromise patient care and result in cardiac arrest [4]. However, these concentrated solutions can be fatal if given inappropriately. Concentrated KCI has been identified as a highrisk medication by organizations in Australia, Canada and the United Kingdom of Great Britain and Northern Ireland (UK). In the United States of America, 10 patient deaths from misadministration of concentrated KCl solution were reported to the Joint Commission in just the first 2 years of its sentinel event reporting programme: 1996–1997. In Canada, 23 incidents involving KCl misadministration occurred between 1993 and 1996 [5–9].

According to a new policy, all hospital wards are now being supplied with commercial “ready-to-use” diluted KCl solutions, which are available in three different concentrations [10]. Concentrated KCl solutions are restricted to the pharmacy and to those critical care areas where concentrated solutions are needed for urgent use such as the Intensive Care Unit, the Cardiac Care Unit and other nominated departments. KCl concentrated solutions have been removed from the routine stock in all other wards and clinical departments.

In emergency cases of severe hypokalemia and in other instances such as heart failure requiring administration of bolus doses, these wards receive concentrated KCl solutions from the pharmacy by personal prescription. Since the hospital pharmacy is not staffed on a 24-h basis, a stock of concentrated KCl is also kept in one of the critical care departments so that it is available to the departments during off-pharmacy hours.

The purpose of this switch was to reduce the potential risk of accidental overdose of IV KCl arising from the use of KCl concentrated solutions by prescribing and using (whenever possible) commercially available ready-to-use diluted solutions, save valuable nursing time, and to ensure that patients requiring IV KCl as part of their treatment continue to receive it promptly and safely.

Switching to KCL dilution solutions is a new national trend emerging in parallel at various hospitals. Several considerations brought us to the decision to choose the FMEA methodology out of a variety of risk assessment methodologies. Firstly, the hospital demanded a prompt assimilation (roughly, in 2 months’ time) of the change. Secondly, since we were dealing with a high alert medication, we looked for a methodology that focuses on systematic identification of the potential risks, prioritizing them and preparing a preventative program in parallel to executing the innovative changes in practice. The FMEA methodology supplied a reasonable solution for the outlined requirements. As different medical centers adopted distinct methodologies and due to lack of accumulative experience in the Israeli healthcare system, we chose a process that enabled us to identify risks and proactively address them before causing any potential harm. The goal of our work was to present a process of risk management as a step toward developing safer hospital policy. The aim of this paper is to present the usage of the FMEA methodology as an advanced tool to achieve this policy. It is worthwhile to emphasize that the FMEA methodology enabled us to make an easy and smooth implementation of a change associated with a high alert drug therapy. In a 4-month time period, most of the physicians and nurses underwent an abrupt change in their therapeutic perception by abandoning a long term and very flexible routine use of KCL solutions and began adhering instead to a strict regimen of only 5 ready to use KCL solutions. Moreover, this transition was implemented without causing any harm to even one single patient.

## Methods

### Failure model effect analysis (FMEA)

FMEA is a systematic technique for failure analysis. Using this tool enables the systematic analysis of postulated component failures and the identification of the resultant effects on system operations [11–13].

FMEA is often the first step of a system reliability study. It involves reviewing as many components, assemblies, and subsystems as possible to identify failure modes, and their potential harm (causes and effects). For each component, the failure modes and their resulting effects on the rest of the system are recorded on a specific FMEA worksheet. The FMEA approach also enables each of the elements comprising a process under investigation to be attributed a cumulative numerical value, the risk priority number (RPN), which can be used to prioritize the action to be taken because it is a numerical rating of the severity, probability and detectability of each failure mode [11–13].

### Conducting the FMEA [14–16]

#### Step 1: selecting the process

#### Step 2: establishing a multidisciplinary action team

In February 2014, a multidisciplinary team including nurses, a pharmacist, physicians, a risk manager, a quality assurance representative and an administrator, gathered in order to analyze the risks of implementing a new policy of using ready-to-use diluted KCl solutions. The team was led by the Deputy Director of the Medical Center.

#### Step 3: mapping the process

**Fig. 1:**
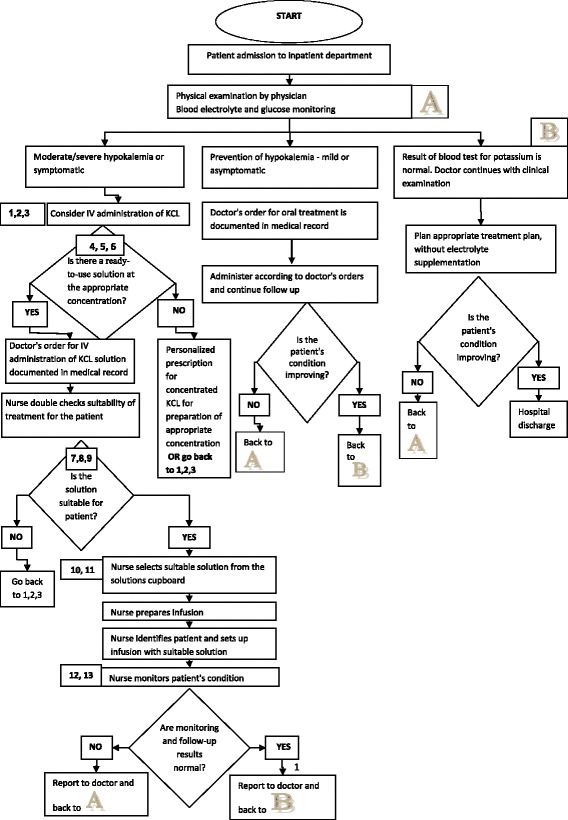
Flowchart of the administration of KCL

After reviewing the current situation, the team identified failure points associated with the implementation of ready-to-use diluted KCl solution, suggested an algorithm and linked a consecutive number to each process step (Fig. 1).

#### Step 4: targeting and listing the possible failure modes

The team identified failure modes associated with the implementation of ready-to-use diluted KCl solutions. The causes of each failure were determined and their impact on patients and/or the organization was defined (Table 1).

**Table 1:** Possible failure modes associated with ready-to-use diluted IV KCl solutions

No	Failure mode	Cause of failure	Effect of failure
1	Error in recorded doctor’s instructions: dose or dosing rate	Inflexibility of the medical staff in adopting new practices	Error in KCL administration due to dose or rate miscalculation
2	Errors in doctor’s instructions documented in medical record: dose or dosing rate	Medical staff fixation on familiar pattern of dosage regimen	Administration of wrong KCL dose
3	Lack of knowledge regarding new and unfamiliar solutions.	Availability of various new and unfamiliar solutions.	Erroneous administration of unsuitable solution.
4	The available new diluted KCL solutions do not meet the patient’s needs and characteristics	KCL solution choice is not one of the 3-standard concentration options	Administration of KCL dose that is unsuitable for the patient
5	The solution vehicle is inappropriate. The infusion itself is incorrect for the patient	Availability of only 2 standard solution vehicles.	Administration of KCL solution that is inappropriate for the patient.
6	The infusion itself is inappropriate for the patient	Lack of ready-to-use KCL solutions in saline 0.45	Administration of KCL solution that is inappropriate for the patient
7	The solution vehicle is inappropriate. The infusion solution itself is inappropriate for the clinical condition of the patient	Ready-to-use dextrose-based solutions may aggravate hyperglycemia in patients on regular diets	Risk of hyperglycemia.
8	The solution vehicle is inappropriate. The infusion solution itself is inappropriate for the patients clinical condition	The new solution may be contraindicated in certain medical conditions	May cause hypernatremia, elevated blood volume, elevated blood pressure and pulmonary edema in patients with fluid restrictions such as those with cardiac and renal insufficiency
9	The solution vehicle is inappropriate. The infusion solution itself is inappropriate for the clinical condition of the patient	Not every solution is appropriate for correction of severe clinical conditions (i.e., treatment of acute severe hypokalemia)	The patient’s emergency status is corrected too slowly
10	Staff is unfamiliar with storage instructions of new solutions	Choice of a broad variety of solutions stored	Solution administration error: administration of a solution without, instead of with, KCL, and vice versa
11	Staff is unfamiliar with storage instructions of new solutions	KCL solutions must be stored separately from non-KCL solutions	Delay in administering the required treatment
12	Lack of knowledge regarding new and unfamiliar solutions	Lack of special handling instructions	Delay in identifying clinical deterioration.
13	Lack of uniform policy regarding administration and frequency of treatment	Lack of policy for use of new solutions	Variation between the various departments in the quality of treatment

#### Step 5: prioritization of the failure modes

Each member of the team conducted and estimated a hazard analysis by prioritizing the failure modes. We prioritized the failure modes identified by using FSP ranking scales:

1. Frequency of occurrence (F)
2. Severity of effects (S)
3. Probability of detection (P).

The product of the amplification of these three scores is the RPN, which represent an index for hazard identification for those modes that pose the greatest potential risks (a higher value represents a greater risk). We decided to initially address the highest rated failure modes and established a “cut–off” RPN value of 300. Table 2 presents all the failure modes identified correlated with their RPN values. The six failure modes chosen and addressed are highlighted in the table. Solutions to the failure modes with the highest ranking may be also be solutions for the less significant failure modes.

**Table 2:** Scoring the failure modes by FSP ranking scales

Failure mode	Frequency of occurrence	Severity of effects	Probability of detection	RPN	Improvement plan
Inflexibility of the medical staff to adopt new practices	10	4	3	120	
Medical staff fixation on familiar dosage regimen	9	4	1	36	
Availability of various new and unfamiliar solutions	10	5	3	150	
KCL solution choice options are limited to only 3 concentrations	9	5	9	405	In emergency situations, when high doses of KCL are needed, the pharmacy will supply solutions containing KCL according to the doctor’s prescription (mEq/100 ml (custom-made medication)
Availability of only 2 types of solution mediums	10	5	9	450	In emergency situations, when high doses of KCL are needed, the pharmacy will supply solutions containing KCL according to the doctor’s prescription (mEq/100 ml)
Lack of pre-prepared KCL solutions in saline 0.45 %	2	5	9	90	
The new solution may be contraindicated in certain medical conditions	10	6	10	600	In emergency situations, when high doses of KCL are needed, the pharmacy will supply solutions containing KCL according to the doctor’s prescription (mEq/100 ml) (custom-made medication)
The solutions are unsuitable for treating acute severe hypokalemia	9	4	9	324	In cases of acute severe hypokalemia, the pharmacy will supply concentrated KCL solutions according to the doctor’s prescription (mEq/100 ml)
A broad variety of solutions in the storage area	4	5	9	324	The pharmacy will distribute precise storage orders
KCL solutions must be stored separately from non KCL solutions	9	8	2	144	
Lack of special handling instructions	7	5	2	70	
Lack of new policy for using the new solutions	10	4	2	80	

*Abbreviations: FSP* frequency of occurrence (F), severity of effects (S), probability of detection (P), *RPN* risk priority number

#### Step 6: development of action plans

The team suggested strategies and developed action plans to be implemented in order to cope with the emerging highest failure modes. The improvement plan is detailed in Table 2. In view of the recommendations suggested, we wrote a protocol for appropriate potassium supplementation use, and distributed it throughout the hospital. We also needed to apply ongoing training of nurses, physicians and pharmacists. This training focused on safety and prevention of potential hazards related to KCl solutions usage.

In emergency situations, when high doses of potassium had to be promptly administered at a high rate in a minimum volume, the physician provided the pharmacy with a personal prescription for concentrated KCl solution, which was supplied to the department as soon as possible. The pharmaceutical services also took a significant role in this procedure by assessing the extent of KCL solution usage of each department and ensuring adequate supplies. In addition, the risk management unit published a warning regarding the use of glucose based KCL solutions in diabetic patients.

The final stage of this FMEA methodology was an evaluation process to examine and validate the implementation of an overall policy, to encourage the use of the ready-to-use commercial solution over the traditional practice.

The evaluation step actually constitutes the conclusive step (step 7) of the FMEA process. Following the implementation of action plans, we sought to look for a measureable method to evaluate the procedure effectiveness. The use of KCl ampoules and ready-to-use solutions post system change was evaluated. As part of the ongoing audit procedure, a periodic (every 3–4 months) 1-day survey was carried out in order to investigate the extent of implementation and adherence to the predefined guidelines for the correct use of the new solutions. This survey sampled 326 patients from medical, surgery, pediatric and intensive care departments. Twenty-eight patients (8.6 %) were intravenously treated with KCl solutions (of any kind).

## Results

FMEA methodology was used to assess the riskiness of each element of the process.

### Ranking

The failure modes for each element were evaluated and ranked according to risk impact and the six primary risks were predefined by the “cut–off” RPN value of 300.

The uppermost impact risk failure modes highlighted in Table 2 are:

1. The new solution may be contraindicated in certain medical conditions.
2. The ready-to-use KCl solutions are only available in normal saline (0.9 %) or in saline 0.45 % + glucose 5 % solutions, which might not always be compatible with the patient’s condition.
3. Ready-to-use dextrose solutions may aggravate hyperglycemia in diabetic patients having regular diets.
4. Ready-to-use solutions are only available in three specific concentrations of KCL.
5. The available concentrations are not suitable for treating acute severe hypokalemia.
6. Storing a broad variety of new solutions in the medicine cabinet may confuse the staff and cause errors upon choosing the right solution.

### Validation

Our observation during the 1-day survey showed that 6 patients out of 28 (21.4 %) were treated with ready-to-use solutions and 22 patients (78.6 %) received tailored personalized prescriptions of KCl solutions (dilution of concentrated KCl solutions) as assessed by their physician. Only 1 patient (3.6 %) out of the 28 treated did not receive the appropriate solution according to the predefined criteria.

### Policy making

Following the FMEA process, survey results and evidence based medicine, hospital guidelines were formulated and published hospital wide.

## Discussion

Medication errors are a huge challenge for many players in the hospital area: clinicians, pharmacists and medical administrators. Historically, KCl solutions were self-prepared and custom-made, mainly due to the high cost involved with the switch. In this article, we describe the FMEA methodology as a smart tool to implement a new policy. The advantages of FMEA as a risk management methodology enable policy makers to identify hazards as a step towards developing safer hospital strategy.

Switching to KCL diluted commercial preparations is a new national trend. As different medical centers embraced their own best practice distinct methodologies, our rationale was to avoid the halo effect when imitating others. Derived from the urge to avoid bias, our strategy was to adopt an analytical methodology for intra- institutional implementation relying on comprehensive considerations of a group of experts within our organization.

**Fig 2:**
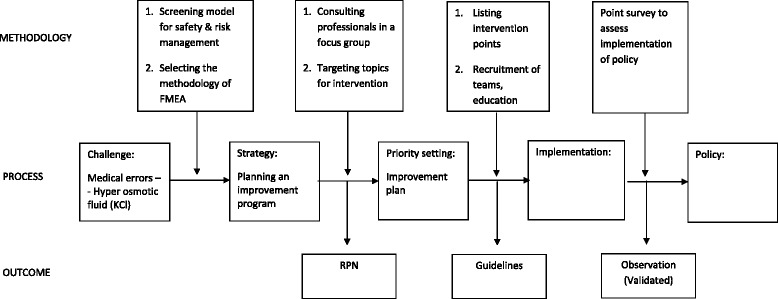
The cascade of methodology- process- outcome

FMEA as an analytical methodology enables in depth inspection to reveal the roots of hazards and take steps towards an acceptable strategy to balance patient safety, effective pharmaceutical activity and economic management. Ranking the different dimensions, the frequency of occurrence, the severity of effects and the probability of detection, clarified the possible loci of intervention paving the way toward desirable directives and cementing organizational policy. The cascade of methodology- process-outcome is presented in Fig. 2. FMEA was used to assess the consequences of implementing a process of substituting concentrated KCl solution with ready-to-use KCl solutions. Out of 13 selected possible failure modes with the highest RPN, six critical major risks were identified. However, only three intervention strategies were required to successfully overcome these obstacles. Using FMEA methodology enabled us to lay out the potential failure modes and focus on the most risky ones. Applying method insights resulted in a remarkable organizational attitude and practice shift towards a top safety level. Our work, similarly to other studies using FMEA methodology [15, 16], showed that FMEA prioritizes the potential harms and minimizes riskiness of complex processes.

The purpose of the 1-day survey was to examine the implementation of the new policy. The results showed adherence to the recommended guidelines and predefined standards in 96.4 % of the cases, supporting a good implementation process, and were compatible with the scoring as was expressed on the FMEA scales. The single inappropriate infusion stated in the results section was associated with a prescribed (out of habit) KCL solution in a concentration the physician used to prescribe before the reform. As a matter of fact, this concentration did not exist amongst the 5 available solutions (though a very similar one did exist). The nurse carried out the written order, though in this specific event, there was no indication to use a concentrated KCL solution at all.

One limitation in our study was that we measured the adherence rates only once, approximately 4 months after the policy implementation starting point. It would be appropriate to examine the adherence to the guidelines again in a 1 year.

## Conclusions

Currently, there is a trend towards switching to commercially diluted KCl solutions as a wise step to minimize the rate of potential medication errors. Before the commencement of the program, several adverse events were reported in our institution. The implementation process has been assessed for a 2-year period, and though the survey was done once, up to date, no incident of adverse event has been reported. This demonstrates clearly the adherence of the medical staff to a presumably, well designed protocol.

Using FMEA methodology enables in-depth inspection to reveal the roots of hazards and take steps towards an acceptable strategy to improve patient safety, effective pharmaceutical activity and economic management. It is an effective proactive risk assessment tool to assist multidisciplinary groups in understanding a process, identifying errors that may occur and reducing potential risks. We have presented a utilization of this method for implementing a change in the routine use of IV KCl. A safe and smooth implementation may encourage us to use FMEA as a universal and generic instrument in future projects at our institution demanding risk minimization.

## Abbreviations

FMEA, Failure mode and effect analysis; FSP, Frequency of occurrence (F), Severity of effects (S), Probability of detection (P); IPSG, International patient safety goals; IV KCl, Intravenous potassium chloride; IV, Intravenous; JCI, Joint Commission International; KCL, Potassium chloride; RPN, Risk priority number
